# Dr. Edward Cresswell Baber

**Published:** 1910-05-28

**Authors:** 


The death is announced of Dr. Edward Cresswell Baber, aged fifty-nine, a former president of the Laryngological Society. Dr. Baber, who was M.B.Lon., L.R.C.P., and M.R.C.S., graduated in 1871, and studied at St. George's Hospital, Paris, and Vienna. He was senior surgeon to the Brighton Throat and Ear Hospital, and formerly a vice-president of the Orological Society.

